# Morphometric Evaluation of the Anterior Meniscofemoral Ligament in Human Cadaveric Knees: Anatomical Variations and Clinical Significance

**DOI:** 10.7759/cureus.79013

**Published:** 2025-02-14

**Authors:** Sidnee Wilson, Annabella Awazi, Trayce Gray, Cameron Cooper, Chakravarthy Sadacharan, Mathew Mendoza, Madeline Ayala, Samantha P Tippen

**Affiliations:** 1 Anatomy, Tilman J. Fertitta Family College of Medicine, Houston, USA; 2 Anatomy, Baylor College of Medicine, Houston, USA

**Keywords:** amfl, anatomy dissection, cadaver, knee, ligament

## Abstract

Introduction

The anterior meniscofemoral ligament (aMFL) is an ancillary ligament within the knee. It has been noted for its rare occurrence and role in stabilizing the lateral menisci. Despite the perceived function, the prevalence of the aMFL is not entirely understood. Additionally, the aMFL holds clinical relevance for practicing physicians when evaluating lateral and posterior knee stability. Thus, this study aims to describe the prevalence of the aMFL and its variation in length and width across genders and knee laterality in all cadavers.

Methods

This cadaveric study evaluated 90 knees in the anatomy lab at Tilman J. Fertitta Family College of Medicine and Baylor College of Medicine. Unpaired T-tests were performed to compare mean variations in length and width between genders and knee laterality. Artificial knees and severely degenerated knees were excluded. Exclusion criteria pertained to samples with a history of osteoarthritis, total knee arthroplasty, or signs of severe tissue degeneration. Dissection was performed using a single transverse cut method with soft tissue extraction. This was done anteriorly with the knee at a 140° flexion angle. Calibrated digital calipers measured length and width.

Results

A total of 90 knees (45 cadavers; 28 women and 17 men) were carefully dissected. The aMFL in 61.76% of male knees (21 out of 34) and 62.5% of female knees (35 out of 56) for a total prevalence of 62.22% across both genders. Unpaired t-tests were conducted to analyze differences between the right and left knees and genders. In males, the right aMFL was found to be significantly wider than the left (p = 0.021). Conversely, in females, the left aMFL was significantly longer than the right (p = 0.0005). There was no significant difference in aMFL width between males and females (p = 0.1573); however, the length of the aMFL was significantly greater in males as compared to females (p = 0.0005).

Conclusion

In conclusion, our study found a high prevalence of aMFL in both sexes, with males having significantly longer aMFL ligaments than females. No significant difference in ligament width was observed between genders. Males had wider aMFL ligaments in the right knee while females had longer aMFL in the left knee. These gender and side-specific variations may impact knee biomechanics and clinical outcomes. Further research is needed to explore the functional implications of these findings.

## Introduction

The knee joint is composed of various structures, in particular, ligaments that play a key role in its functionality and stability. The commonly discussed ligaments are the cruciate and collateral ligaments, which function during flexion and extension as well as during lateral and medial resistance, including maneuvers involving torque. Meniscofemoral ligaments have more recently begun to be investigated, as they hold great potential for their ability to provide additive stability to the knee joint. The anterior meniscofemoral ligament (aMFL), also known as the ligament of Humphrey, more specifically is of interest due to its rarity. Furthermore, its function is similar to some of the functions of the anterior cruciate ligament (ACL) in that it is taut during knee flexion, indicating potential roles in knee stability during this type of motion [1,2].

Surprisingly so, though similar in its stability function, the location of the aMFL is distinctly different from that of the ACL with it falling anteriorly parallel to the posterior cruciate ligament (PCL) having an origin and insertion on the posterior horn of the lateral meniscus and anteriorly on the medial femoral condyle, respectively [3,4]. With this in mind, it may add to torque stability and has been proposed to have roles in the stability of the lateral knee compartment as well as the maintenance of the anatomic positioning of the interface of the tibia and fibula [4]. This is most promising with the consideration that knee pain is among the most common reasons for orthopedic visits in the United States and that ACL tears are the number one injury of the knee seen by physicians, comprising approximately 400,000 surgeries each year in the United States [4,5]. Furthermore, in patients with ACL deficiencies, there has been an increased progression toward the development of arthritis and injuries to the menisci and chondral structures [5].

In a review of the literature, though limited, studies varied in their approach, with some using cadaveric, arthroscopic, and imaging-based approaches to understand the aMFL. The prevalence widely varied across studies [2-4,6]. Additionally, the aMFL was notably more difficult to visualize in imaging studies. Though not as successful as cadaveric or arthroscopic studies, this ligament has been located using 3T and T2 WI MRIs [2,4,6]. Other limitations found in prior studies include sample biases that limit generalizability; the population studied included solely patients with pre-existing knee problems who were seeking treatment [2]. Additionally, there have been concerns, as it pertains to the generalizability of other demographic factors such as age. Race/ethnicity and unequal number of left and right legs were used for analysis [2,4]. Of the cadaveric studies researched, they proved limited in their exploration of the aMFL and associated demographic data.

Understanding aMFL as it pertains to its location and dimensions will help expand upon the understanding of its function. Further consideration of age and gender relating to the aMFL are important as well; they show potential to further insight into their impact on hormones, composition changes, and ability to reflect anatomic wear and tear [5]. Understanding these and biological factors like height may be key variables in relation to prevalence, overall ligament strength, and knee stability.

The objective of this inclusive cadaveric dissection-based study is to further investigate and contribute to the limited data on the aMFL to further understand the anatomic positioning to guide MRI identification of this ligament, and further understanding of its size, gender occurrence, and anatomic position without the limitations of investigation based on known knee pain. It also aims to explore correlations in measurement and prevalence of the aMFL in association with gender factors to guide future practice protocols, as it pertains to the prevention and treatment of knee pain and injuries to improve patient satisfaction and quality of life.

## Materials and methods

Study design

A total of 90 included knees (45 cadavers; 28 (62.2%) women and 17 (37.78%) men) were carefully dissected in the anatomy lab with a specific anterior dissection technique at Tilman J. Fertitta Family College of Medicine and Baylor College of Medicine. The ages of the cadavers ranged from 46 to 93 years.

Inclusion and Exclusion Criteria

Of the total 95 knees, 5 were excluded, 3 males and 2 females, due to previously undergone knee replacement surgery. Samples with a history of osteoarthritis, total knee arthroplasty, or any signs of severe tissue degeneration were also excluded. The knee samples were not previously dissected and had all relevant structures intact.

Data collection

To view the aMFL, a transverse cut was made across the patellar tendon just above the tibial tuberosity. Then, the knee was fully flexed with slight external rotation, and additional transverse cuts were made along the medial and lateral aspects of the proximal tibia to allow full access to the joint space. The ACL, as well as superficial soft tissue, including intra-articular adipose tissue, synovium, and synovial fluid, was removed in order to visualize the PCL. Meniscal components were carefully avoided to maintain proper tension and accurate attachment points of the aMFL. If present, the aMFL was visualized by fibers traveling obliquely along the PCL from the medial portion of the intercondylar fossa of the femur to the posterior horn of the lateral meniscus.

The length of the aMFL was measured from the aMFL and pMFL divergent point from the posterior horn of the lateral menisci to its origin point within the intercondylar fossa of the medial surface of the lateral femoral condyle. The measurements for width were taken at an approximate midpoint along the ligament. All measurements were taken using calipers. No significant Interobserver measurement variations were noted. Each measurement for both length and width was taken twice within every knee. Subsequently, the measured values were summed and then divided by two, yielding an individualized average for each specific knee. This study is IRB-exempt under federal regulations.

Statistical analysis

These averages were recorded and graphically represented using Microsoft Excel (Microsoft Corporation, Redmond, WA, US). Values were calculated using GraphPad PRISM 10.2.0 software (GraphPad Software, Inc., San Diego, CA, US).

## Results

**Figure 1:**
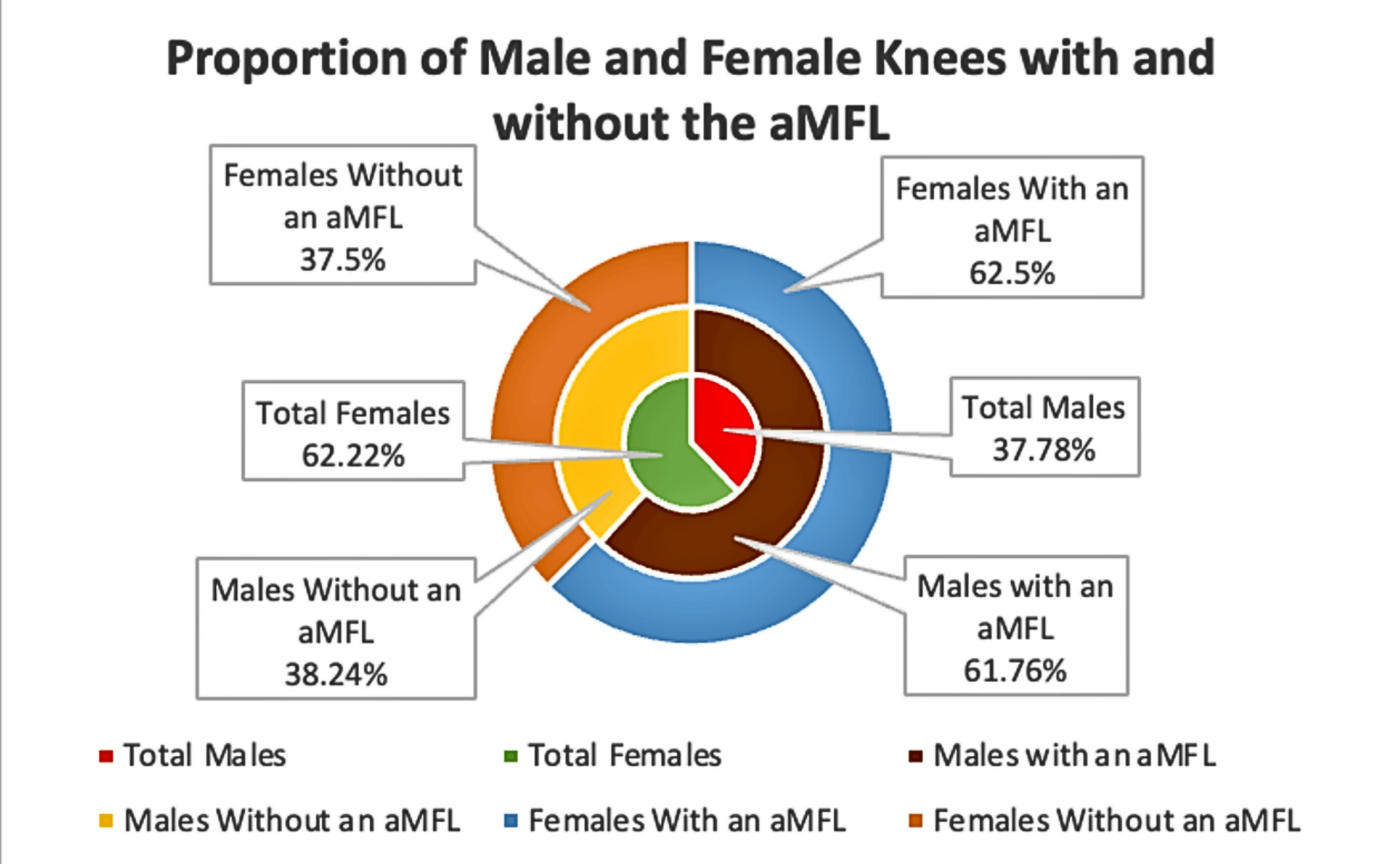
Prevalence of the aMFL as specified by gender and total aMFL: anterior meniscofemoral ligament

An aMFL was observed in 21 out of 34 (61.76%) male knees and 35 out of 56 (62.5%) female knees for a total prevalence of 62.22% across both genders (Figure 1). These findings suggest the aMFL was found In the majority of the sample population overall.

Unpaired t-tests were conducted to analyze differences between the right and left knees and genders. In males, the right aMFL was found to be significantly wider than the left (p = 0.021) (Table 1). Conversely, in females, the left aMFL was significantly longer than the right (p = 0.0005) (Table 1). Significance was determined at a p-value of p=<0.05.

**Table 1:** Summary of the data, including the mean, range, standard deviation, and p-value for male and female knees, further categorized by left and right knees P-values calculated through the unpaired T-test

Width							Length				
		Mean	Range	Std. Deviation	P-Value	T-Value	Mean	Range	Std. Deviation	P-Value	T-Value
Male	Right	6.48	2.02	0.91	0.021	T=1.1	30.97	8.77	3.34	0.507	T=0.8
	Left	5.13	4.41	1.64			28.84	14.65	5.1		
Female	Right	5.16	4.73	1.36	0.577	T=0.7	23.74	9.43	2.7	0.0005	T=1.4
	Left	5.38	5.93	1.65			26.81	11.29	3.34		

**Figure 2:**
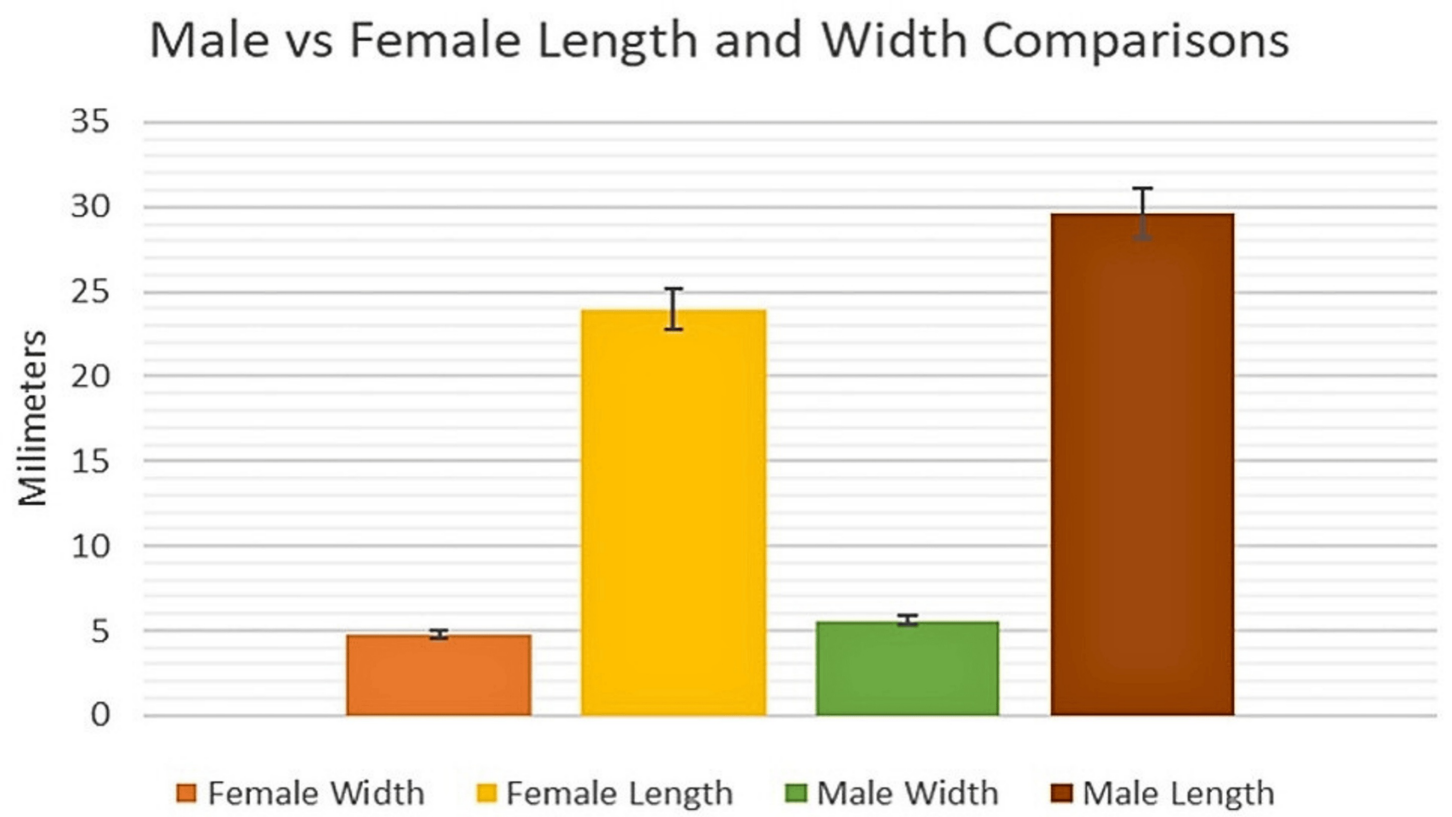
Comparison of the length and width of the aMFL between genders On average, the length of the AMFL is greater in males compared to females while the width remains relatively consistent across genders. aMFL: anterior meniscofemoral ligament

While there was no significant difference in aMFL width between males and females (p = 0.1573) (Figure 2), the length of the aMFL was significantly greater in males as compared to females (p = 0.0005) (Figure 2). This finding suggests gender-influenced discrepancies between aMFL lengths when comparing males to females.

**Figure 3:**
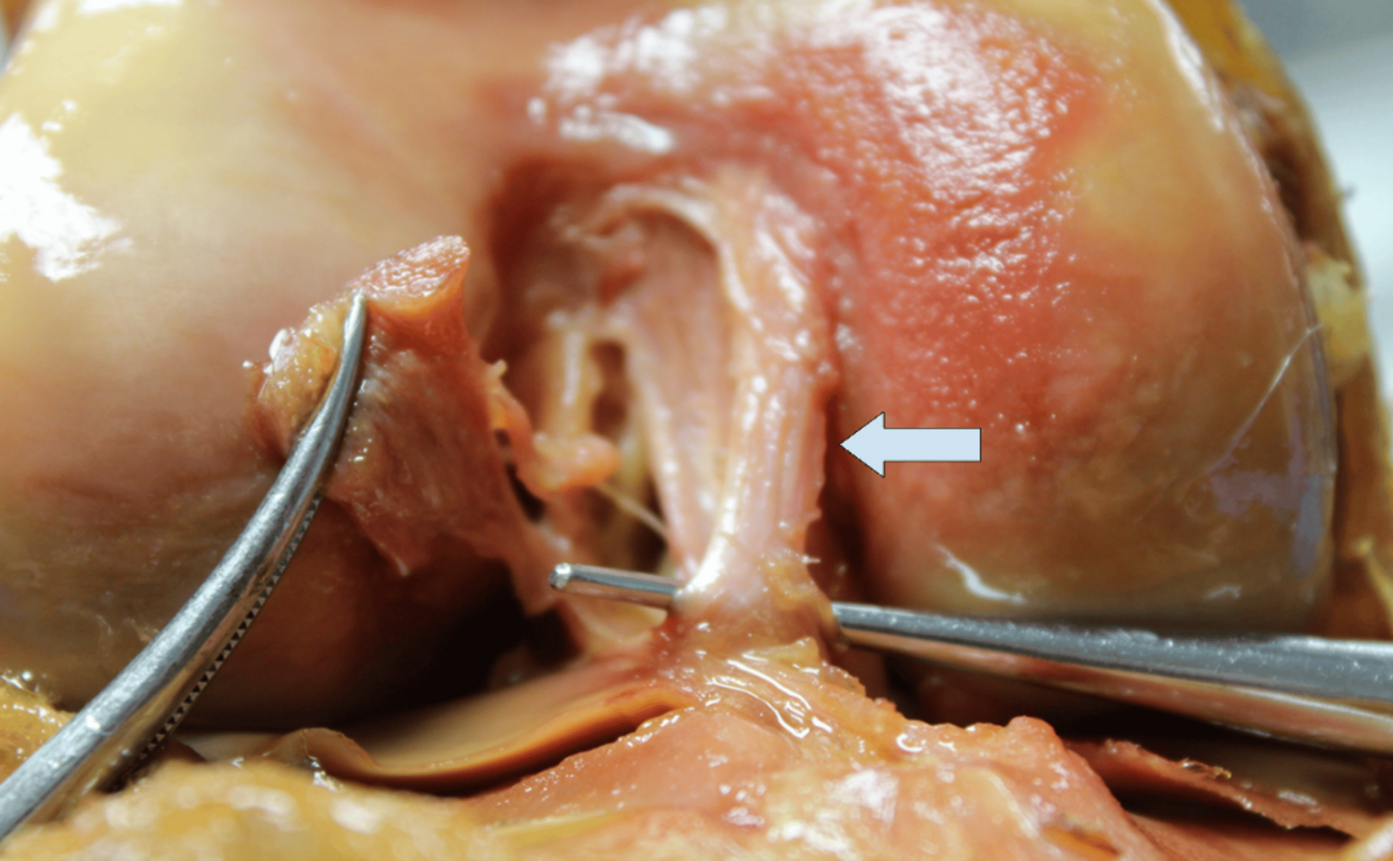
aMFL highlighted with probe Measurements were taken using digital calipers inferior to the arrow at the origin - posterior horn of the lateral meniscus, and insertion - medial aspect of the lateral femoral condyle. aMFL: anterior meniscofemoral ligament

Shown below is the aMFL (Figure 3), which originates from the anterior horn of the lateral meniscus and inserts onto the medial surface of the femur at the intercondylar notch.

## Discussion

As the scope and expansion of clinically relevant anatomical knowledge become more prominent in anatomical literature, gaps in our knowledge and understanding become more apparent. The anterior meniscal femoral ligament (aMFL) or Humphrey's ligament is one of the gaps in knowledge. To date, there is limited literature examining morphological variation, including length, width, and prevalence. This study aimed to analyze the morphological changes found in cadaver specimens and compare them with existing literature. Although cadaveric studies are limited, a recent meta-analysis detailed a 55.5% aMFL prevalence across studies totaling 4220 limbs [2]. This is a stark contrast to previous literature done in smaller studies, which detailed a sample size of 26 with an aMFL prevalence of 17.4% [7,8]. Given the trivial nature of the prevalence reporting of studies, there is no surprise at the immense scientific interest surrounding the anterior meniscofemoral ligament. In our study, the aMFL was present in 62.22% of dissected specimens, suggesting a marginal, but potentially significant, higher prevalence of this ligament than previously suspected.

A higher prevalence of the aMFL could implicate several things. First, a higher prevalence may indicate the ligament’s sheer necessity in its proposed functionality. Specifically, stabilizing the lateral meniscus. According to a recent study, the aMFL has a pronounced role in stabilizing the posterior-lateral meniscus in ACL-deficient knees [9]. In contrast, earlier studies of aMFL functionality highlight its role in stabilizing the posterior cruciate ligament [9].

Our study has identified limitations pertinent to it. These limitations may have impacted the final data output of this study. First, we have identified the potential effect of sample size on our study. This study examined 90 total cadaveric specimens. In future studies, the sample size should be increased to improve the power of the study. Second, differences in dissection procedures may affect the measurement of various variables presented within the data. Additionally, dissection variability and exclusion of degenerative knees may have played a role in our results. 

Our recommendations for future studies to overcome these possible limitations include implementing a standardized dissection procedure for each ligament and increasing our sample size. Standardized procedures for ligament extraction and exposure can help reduce inevitable errors and operator variability. Additionally, having a higher availability of cadaveric specimens could provide a more robust and variable sample population and improve extrapolation to the global population. This could increase the power of the study and its generalizability to outside contexts. These recommendations, although interesting, are not all-encompassing to this study's limitations.

## Conclusions

This study provides important insights into the anatomical characteristics of the anterior meniscofemoral ligament (aMFL) in both male and female populations. Our findings suggest that the aMFL is a common anatomical feature although its presence and characteristics vary slightly by gender. Significant differences were observed in aMFL length, with males having significantly longer aMFL ligaments compared to females However, no significant disparity in width was found between ligaments when comparing genders. Males presented significantly wider aMFL ligaments in the right knee compared to the left. In females, the left aMFL was significantly longer than the right. This study highlights both gender and side-specific anatomical variations in the aMFL, which have implications for knee biomechanics, meniscal stability, and potential clinical outcomes. Future research to expand on our findings on a larger scale with more robust demographics is needed to confirm our findings. 
